# Bilateral Giant Cell Tumour of the Achilles Tendon Sheath: A Rare Case Managed with Peroneus Brevis Tendon Transfer

**DOI:** 10.1155/cro/4213959

**Published:** 2026-07-02

**Authors:** Yasir Hidayat, Obaid Ur Rehman, Muhammad Mobushir, Osama Ijaz

**Affiliations:** ^1^ Department of Orthopedics, Benazir Bhutto Hospital, Rawalpindi, Pakistan; ^2^ Department of Orthopedics, Rawalpindi Teaching Hospital, Rawalpindi, Pakistan

**Keywords:** achilles tendon, case report, gctts, peroneus brevis, tenosynovial giant cell tumor

## Abstract

**Case:**

A 34‐year‐old male presented to the outpatient department with bilateral posterior ankle swelling. X‐ray imaging showed no bony involvement, but histopathology was suggestive of bilateral giant cell tumor of the Achilles tendon. Both lesions were excised with meticulous dissection and transfer of peroneus brevis tendon. Postoperative recovery was uneventful, with favorable short‐term functional outcome.

**Conclusion:**

This rare case of bilateral giant cell tumor of the Achilles tendon highlights the importance of early recognition and careful planning of reconstructive approaches to ensure optimal outcomes while minimizing functional deficits.

## 1. Introduction

Giant cell tumor of the tendon sheath (GCTTS) is a benign, solitary, localized, painless, slow‐growing extra‐articular mass [1] arising from the synovium of tendon sheaths, bursae, or joints and commonly affects females [2]. The lesion typically affects adults aged 30–50 years and is more prevalent in the hand, second only to ganglion cysts among benign soft tissue tumors [3]. Its occurrence in the foot and ankle is uncommon [4].The Achilles tendon is the body’s longest and strongest tendon, formed by the convergence of the gastrocnemius and soleus muscles and inserting onto the calcaneus. The incidence of GCTTS is approximately 4 cases per million person‐years, with bilateral involvement of the Achilles tendon being exceptionally rare [5]. GCTTS is a locally aggressive lesion which is capable of causing progressive destruction of adjacent soft tissues. When it involves Achilles tendon, it leads to tendon degeneration through direct invasion and by compromising its vascular supply [6, 7]. Progressive enlargement of the tumor over the Achilles tendon may result in both functional impairment and cosmetic deformity, with a small risk of malignant transformation. Surgical excision remains the primary treatment modality; however, its repair or reconstruction of the resultant defect may require tendon transfer or graft augmentation. Multiple reconstructive techniques have been described but the choice and functional outcome is largely dependent on size of the defect and the tendon selected for transfer [8]. We present a rare case of bilateral GCTTS of the Achilles tendon managed with excision and reconstruction using peroneus brevis tendon transfer. This case report was prepared and reported in accordance with the CARE (CAse REport) guidelines.

The patient was informed of the plan to publish this case, along with pertinent clinical information and photographs, to assist in medical knowledge. Written patient informed consent was granted for publication of this report and accompanying photographs, with an assurance that his identity would be kept confidential. The patient is still being followed up postoperatively for functional outcome as well as possible complications if any.

This study was conducted in accordance with institutional ethical standards, and formal ethical approval was waived for this single case report.

## 2. Case Report

A 34‐year‐old male driver by profession and had no significant past medical/surgical history of any kind. He presented with swelling over the posterior aspect of the ankle for the last 1 year, without any history of trauma. The swelling was spontaneous in origin, gradually increased over time, and was initially painless but later became painful, limiting the patient’s daily activities. On clinical examination, both swellings were globular, smooth in texture, firm in consistency, non‐fluctuant, mildly tender on deep palpation, non‐erythematous, and without any overlying skin changes. On the right side, it measured approximately 5 × 4 cm in size, while on the left side it measured 2 × 2 cm.

Ultrasound showed features of Achilles tendinopathy with suspicion of an Achilles tear. MRI was performed, which demonstrated thickening of the distal Achilles tendon with associated soft tissue thickening. **The lesion appeared isointense on T1-weighted images and heterogeneously hyperintense on STIR/PD fat-saturated sequences, with heterogeneous post-contrast enhancement. On Gradient echo (GRE) sequences, no areas of blooming are seen and Achilles tendon appears intact**. Findings were suggestive of a tenosynovial proliferative lesion, with differentials including giant cell tumor of the tendon sheath, para‐tendinous fibrosis and synovial sarcoma.

To confirm the diagnosis, histopathological analysis was performed via trucut biopsy of the lesion, which showed **multiple multinucleated osteoclast-type giant cells along with numerous foamy histiocytes. Mononuclear medium sized tumor cells were noted with indistinct nucleoli and occasional mitotic figures. There were extensive areas of cholesterol cleft formation with associated foreign body type giant cell reaction. CD-68 highlights histiocytes, multi-nucleated giant cell as well as mononuclear tumor cells. The overall picture was suggestive of tenosynovial giant cell tumor.** Following the results of all preliminary investigations, surgical excision was planned. Pre‐anaesthesia assessment and baseline workup were unremarkable. Surgery was planned with excision of the lesion and reconstruction using transposition graft of the peroneus brevis tendon.

**Figure 1:**
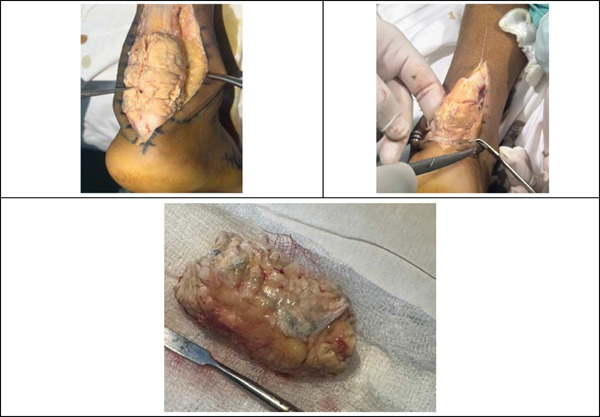
Intraoperative exposure of bilateral Achilles tendon lesions and excised specimens.

**Figure 2:**
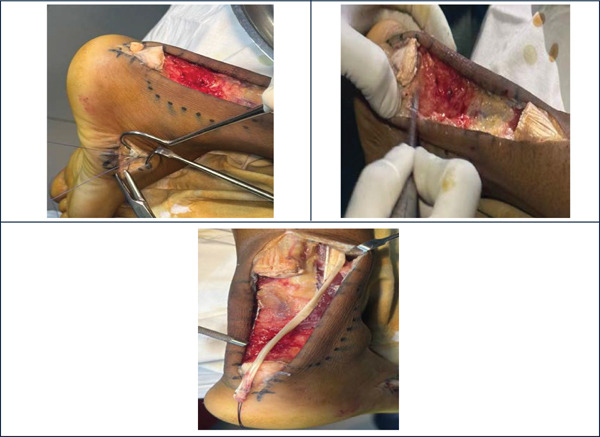
Harvesting of peroneus brevis tendon through lateral incision.

The patient was approached through a posteromedial incision over the Achilles tendon. Yellowish‐white masses were exposed bilaterally along their entire length and excised (Figure 1). They had a smooth texture, with dimensions of 5 × 4 cm on the right side and 2 × 2 cm on the left side. The residual defect created was repaired using transposition grafts of the peroneus brevis. The peroneus brevis tendon was approached through a linear incision over the base of the 5th metatarsal and mobilized to be placed at the site of the defect (Figure 2). The lengths were adjusted such that both tendons had equal tension.

The selection of the peroneus brevis tendon was further supported by its anatomical and functional compatibility with the Achilles tendon. The tendon lies in close proximity and shares a similar line of pull and excursion, allowing effective restoration of plantarflexion. Additionally, it provides sufficient length and tensile strength to bridge large defects, while its harvest results in minimal donor‐site morbidity due to compensatory function by the peroneus longus.

**Figure 3:**
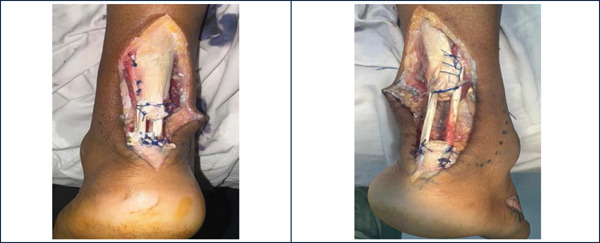
Reconstruction using Pulvertaft weave technique.

**Figure 4:**
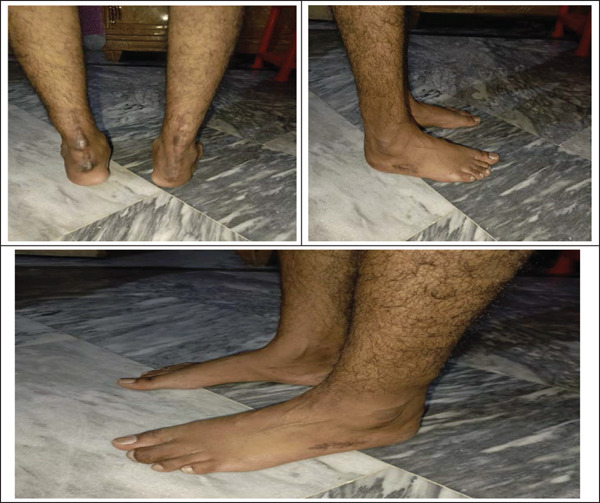
Clinical outcome at 6‐month follow‐up.

Closure was performed using the Pulvertaft technique (Figure 3), and the foot was placed in a long leg equinus backslab for 2 weeks (Table 1). This was converted to an above‐knee cast at 2 weeks after stitch removal and wound assessment, and maintained for 4 weeks. Subsequently, equinus was gradually corrected to a neutral position by adjusting the cast at a rate of 10° dorsiflexion per week. This process was completed over a 2‐month period, after which the above‐knee cast was converted to a below‐knee cast. The foot was maintained in a neutral position, and weight bearing was initiated. After a further 2 weeks, the below‐knee cast was removed, and ankle physiotherapy along with full weight bearing was started to restore full range of motion. Functional outcomes were assessed at each follow‐up, and at 6 months, evaluation using the AOFAS score demonstrated favorable functional outcome with no functional impairment till early follow‐up visits (Table 2, Figure 4).

**Table 1:** Timeline of post‐operative immobilization and rehabilitation following tendon reconstruction.

Rehabilitation Protocol	
0–2 weeks	Equinus backslab
2–6 weeks	Above‐knee cast
6–10 weeks	Gradual dorsiflexion (10°/week)
10–12 weeks	Below‐knee cast + weight bearing
>12 weeks	Cast removal + physiotherapy

**Table 2:** AOFAS ankle–hindfoot score pre‐ and post‐operatively.

Parameter	Maximum Score	Pre‐Op Score	Post‐Op Score
Pain	40	25	40
Function	50	35	45
Alignment	10	7	10
**Total**	**100**	**62**	**95**

It is acknowledged that the AOFAS ankle‐hindfoot score, while widely used, serves as a functional proxy rather than a comprehensive patient‐reported outcome measure, and has recognized limitations including examiner variability and ceiling effects. In addition to AOFAS scoring, functional assessment at six months included evaluation of range of motion, gait analysis, and muscle strength testing. The patient demonstrated full plantarflexion and dorsiflexion bilaterally, a normal gait pattern with no observable asymmetry, and muscle strength of 5/5 on both sides. The patient expressed complete satisfaction with the surgical outcome and reported no functional limitation in daily activities.

### 2.1. Patient Perspective

The patient reported high satisfaction with the surgical outcome, noting significant improvement in pain and ability to perform daily activities. He expressed confidence in returning to routine work without limitation and was satisfied with both functional and cosmetic outcomes.

## 3. Discussion

Giant cell tumor of the Achilles tendon is primarily a tumor which surrounds tendon sheaths. The rare occurrence of the lesion, its painless nature, and progressive increase in size make it prone to misdiagnosis and sometimes neglect [1]. This case highlights that giant cell tumor (GCT), although rare, should be considered as a differential diagnosis when evaluating ankle swelling, and a multidisciplinary approach may be required for optimal management. Our case adds to the existing knowledge on this rare entity, as no standard guidelines are currently in place.

Prior studies have managed such cases with various techniques. Nirmal C [8], in a case series of three patients, managed the condition using excision and transposition of tendons of peroneus brevis (PB) and tibialis posterior (TP). This study demonstrated satisfactory results similar to ours; however, we utilized only a single tendon (PB). In another study conducted in Wenzhou, China, a similar lesion was treated with excision and hydrogen peroxide lavage, and the defect was repaired using autografts of semimembranosus and semitendinosus on the right side and flexor hallucis longus tendon on the left side [9]. Samal P [10] reported a similar technique using dual tendon transfer, where both tendons were mobilized and fixed to the calcaneum.

Previous studies also suggest that defects greater than 5 cm should be treated with tendon transfer. Maffulli et al. described that peroneus brevis tendon transfer is a less invasive and effective option for large Achilles tendon defects, with good functional outcomes [11]. In a systematic review, it was also noted that local tendon transfers such as flexor hallucis longus and peroneus brevis provide favorable clinical results [12]. Bosworth and Garabito described alternative techniques using gastrosoleus turn‐down flaps [13]˒ [14]. Wapner et al. suggested that flexor hallucis longus transfer is a viable alternative; however, rehabilitation may be more challenging due to its relatively smaller muscle belly and functional differences compared to the Achilles tendon [15].

The choice of peroneus brevis tendon in our case is supported by several biomechanical and anatomical advantages. Its proximity to the Achilles tendon allows harvesting through a single incision, reducing operative time and soft tissue dissection. Functionally, the peroneus brevis has a line of pull and excursion similar to the Achilles tendon, facilitating effective restoration of plantarflexion. Biomechanically, it possesses adequate tensile strength to withstand physiological loads, making it suitable for reconstruction of large defects. Furthermore, donor‐site morbidity is minimal, as the peroneus longus compensates for eversion, thereby preserving overall foot biomechanics. In contrast, alternative tendons such as flexor hallucis longus or tibialis posterior may alter gait mechanics or compromise medial arch stability. These factors, along with anatomical proximity to the Achilles tendon and synergistic action with the gastrosoleus complex, make it a suitable candidate for tendon reconstruction. Its transposition increases the lever arm for plantarflexion, providing a biomechanical advantage and facilitating rehabilitation without significant functional loss.

All the previous literature describes various approaches to this unique condition, and due to its rarity, no standardized protocol exists. In our case, we observed that using a single tendon, securing it via the Pulvertaft technique, and subsequent immobilization in a cast resulted in favorable functional outcomes without impairment. A recent case report by Kumar et al. further supports the importance of individualized surgical planning and careful outcome interpretation in rare tendon sheath tumors, reinforcing that meticulous excision combined with appropriate tendon reconstruction yields favorable short‐term functional results in such uncommon presentations [*16* *]*. However, long‐term outcome data remain limited in such rare presentations.

Recurrence following surgical excision of GCTTS remains a well‐recognized concern. For the localized subtype, recurrence rates in the literature range from 10% to 20%, while the diffuse subtype, as seen in our case, carries a considerably higher recurrence risk of up to 40–50%, largely attributed to its infiltrative growth pattern and the difficulty of achieving clear surgical margins. Bilateral involvement, as seen in our patient, further compounds this risk, as both operative sites require independent monitoring and any recurrence on either side may compromise overall functional recovery. Given that our patient remained asymptomatic at six months with no clinical evidence of recurrent swelling on either side, we elected to continue clinical surveillance rather than routine imaging. Follow‐up was structured around regular outpatient review with thorough clinical examination of both ankles. Imaging in the form of ultrasound or MRI was reserved for cases where clinical suspicion of recurrence arose, in keeping with a symptom‐guided approach. We believe this pragmatic strategy is appropriate in resource‐limited settings, while acknowledging that imaging‐based surveillance may offer earlier detection of subclinical recurrence.

At the end, we suggest that this approach may be considered for managing bilateral GCTTS, as it provides satisfactory postoperative outcomes, comparable to previously reported studies [8].

One limitation of this report is the relatively short follow‐up duration of six months. While early functional outcomes were favorable, longer follow‐up is necessary to draw definitive conclusions regarding durability of reconstruction and late complications.

The key lessons which we can draw from this case are that bilateral GCTTS of the Achilles tendon, though rare, should be considered in young patients presenting with bilateral posterior ankle swelling, and that surgical technique of using peroneus brevis tendon transfer with the Pulvertaft technique is a reliable single‐tendon reconstructive option with satisfactory early functional outcomes.

## 4. Conclusion

This case of bilateral giant cell tumor of the Achilles tendon highlights the importance of early recognition and careful planning of the reconstructive approach. Short‐term follow‐up demonstrated favorable functional outcomes. It is acknowledged that the follow‐up period of six months is limited, and extended surveillance will be required to confirm the durability of these outcomes and potential complications if any.

## Funding

No funding was received for this manuscript.

## Conflicts of Interest

The authors declare that they have no conflicts of interest regarding the publication of this case report.

## Data Availability

The data that support the findings of this study are available from the corresponding author upon reasonable request.
